# Percutaneous cannulated screw fixation for pediatric epiphyseal ankle fractures

**DOI:** 10.1186/s40064-016-3623-1

**Published:** 2016-11-07

**Authors:** Özgür Çiçekli, Güzelali Özdemir, Mustafa Uysal, Vedat Biçici, İzzet Bingöl

**Affiliations:** 1 Department of Orthopaedic Surgery and Traumatology, Sakarya Training and Research Hospital, Sakarya, Turkey; 2 Department of Orthopaedic Surgery and Traumatology, Numune Training and Research Hospital, Ankara, Turkey; 3 Department of Orthopaedic Surgery and Traumatology, Sakarya University School of Medicine Training and Research Hospital, Sakarya, Turkey; 4 Department of Orthopaedic Surgery and Traumatology, Yenimahalle Training and Research Hospital, Ankara, Turkey; 5 Department of Orthopaedic Surgery and Traumatology, 29 Mayıs State Hospital, Ankara, Turkey

**Keywords:** Physeal fractures, Ankle, Cannulated screw, Headless compressive screw, Physeal arrest

## Abstract

**Background:**

Ankle injuries are among the most common injuries in children. The aim of this study was to compare the efficacies of two percutaneous fixation methods after closed reduction in physeal ankle fractures.

**Methods:**

We reviewed the cases of 24 patients with a mean age of 12.29 years; 16 were male, and 8 were female. Only patients with fractures of Salter-Harris types 2, 3, and 4 with displacements greater than 2 mm were included in the study. Patients were treated with closed reduction manipulation and percutaneous screw fixation. For each patient, either cannulated or headless full threaded compressive screws were used for percutaneous fixation. Radiological and clinical healing time, range of motion (ROM), American Orthopaedic Foot and Ankle Society (AOFAS) score and physeal arrest were then measured.

**Results:**

The mean follow-up time was 13 months. The mean time until cast removal was 3.5 weeks (range 2–5). A full ROM was achieved at an average of 5.7 weeks postoperatively (range 4–8). The radiologic healing time was 6.1 weeks (range 4–7). The patients’ clinical healing time averaged 6.8 weeks (range 5–8). Differences in radiologic healing time (p = 0.487), clinical healing time (p = 0.192), AOFAS score (p = 0.467), and complication rate (p = 0.519) between patients who received the headless compressive screw and those who received the cannulated screw for fixation were not statistically significant.

**Conclusions:**

We demonstrate good clinical results with closed reduction and the percutaneous screw fixation method. Both cannulated and headless compressive screws can be used safely as a treatment method in physeal ankle fractures.

## Background

In children, the physes tend to be more susceptible to damage than the surrounding tissues. Traumatic ankle injuries are more likely to cause injury to the physes or bone than to ligaments during childhood (Wuerz and Gurd 2013). Ankle injuries are very common in children (Podeszwa et al. 2008) and are second only to wrist and hand injuries in children between the ages of 10 and 15. Ankle fractures account for approximately 5% of pediatric fractures and 15% of physeal injuries (Mizuta et al. 1987; Peterson and Peterson 1972; Worlock and Stower 1986). The distal tibial physis accounts for 45% of tibial growth (Wuerz and Gurd 2013). When treating children with physeal ankle fractures, it is important to achieve a satisfactory reduction and avoid physeal arrest to minimize the risks of angular deformity, early arthrosis, leg length inequality, and joint stiffness (Wuerz and Gurd 2013; Kay and Matthys 2001).

Acute pediatric ankle fractures can be treated by closed reduction and casting. For cases in which satisfactory reduction is not achieved through these procedures, previous studies, have applied the treatment modalities of open reduction and internal fixation (ORIF) (Lintecum and Blasier 1996; Dias and Giegerich 1983; Gönç and Kayaalp 2004; Kling et al. 1984). Here, we sought to investigate the efficacy of two percutaneous fixation methods after closed reduction in physeal ankle fractures. We achieved similar results in physeal ankle fractures regardless of the use of standard cannulated screws (3.5 mm) or headless compressive cannulated screws (3 mm) for fixation.

## Patients and methods

We identified 56 patients with physeal ankle fractures, and 24 met our inclusion criteria. Only patients with fractures with displacements of greater than 2 mm of Salter-Harris type 2, 3, and 4 were included in the study. Salter-Harris type 1 and 5 fractures, nondisplaced, and undiagnosed fractures were excluded. Fractures with callus formation lasting longer than one week after trauma were classified as undiagnosed fractures and were also excluded. Patients treated with a brace were not evaluated. Only patients who required closed reduction and surgical fixation were included in the study.

The 24 patients who met the inclusion criteria ranged in age from 8 to 15 years with a mean age of 12.29 years. In total, 16 were male, and 8 were female. The physical examination focused on skin defects, swelling, neurologic deficits, vascular injury, and deformity. AP (anteroposterior) mortise and lateral radiographs of the patients were obtained during the first examination. Closed reduction and casting were performed in the emergency room without anesthesia. Ankle radiographs were also obtained after casting. Fractures that appeared unreduced in the X-rays were treated surgically. Computed tomography (CT) was used to further image all the patients before their surgeries to determine the size and shape of the fracture fragments and to measure the displacement, the intra-articular gap, and the step-off.

**Fig. 1:**
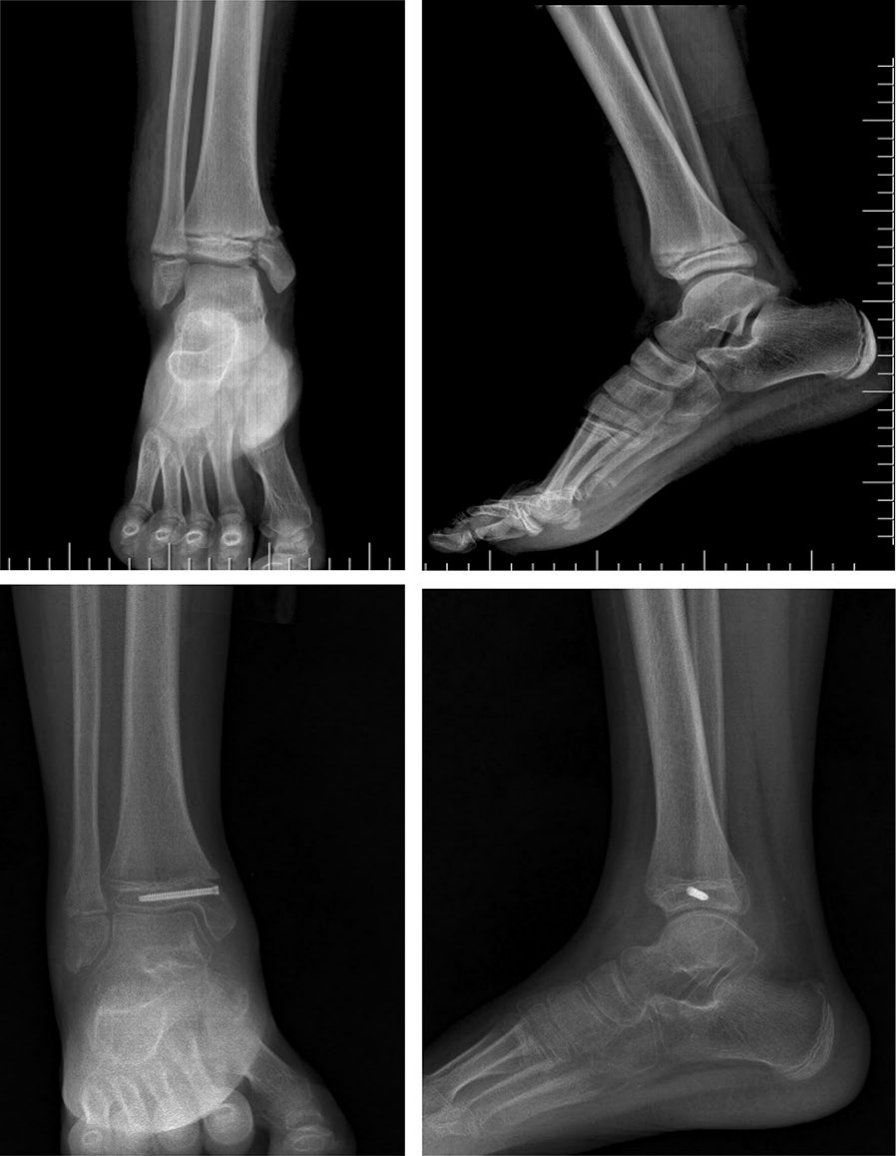
A 12-year-old boy with Salter-Harris type 4 physeal fracture. Pre-operative and post-operative X-rays after 3 months

**Fig. 2:**
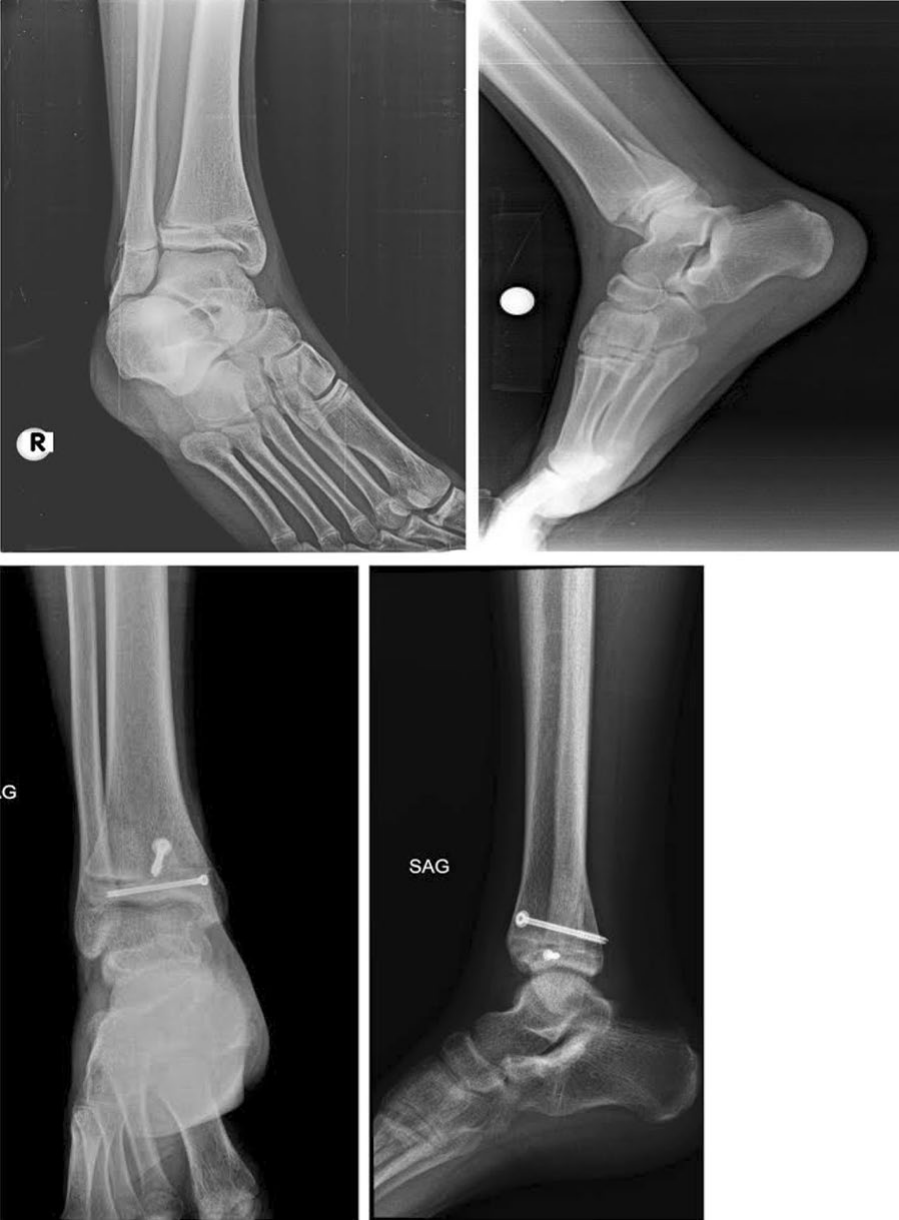
A 14-year-old boy with Triplane fracture. Pre-operative and post-operative X-rays after 3 months

Patients were treated using closed reduction and percutaneous cannulated screw fixation under general anesthesia in the first 24 h after trauma. Closed reduction was achieved with small Kirschner wires (K-wires) under fluoroscopy. The K-wires were used to reduce the displaced fragments into the anatomical position. A guiding K-wire was placed perpendicular to the fracture line and parallel to the physeal line. A cannulated drill was used to drill the cortex onto the guide wire. Two types of screw were used for percutaneous fixation: cannulated screws (UniCan, Ortopro, Turkey) and headless full threaded compressive screws (Acutrak, Acumed, USA) (Figs. 1, 2). The screw type used in each operation was selected randomly. Step-off and gap compression after reduction and fixation was assessed using fluoroscopy. Additional distal fibular physeal fractures were reduced and fixed with K-wire during the same operation.

Short leg casts were applied after the operations. Each patient began ankle range of motion (ROM) exercises after one week. Patients were examined every week at an outpatient clinic. Fracture healing was approved after callus formation on AP and lateral ankle radiographs (digital x-ray system). Patients were allowed to bear weight on their affected ankles after callus formation.

**Fig. 3:**
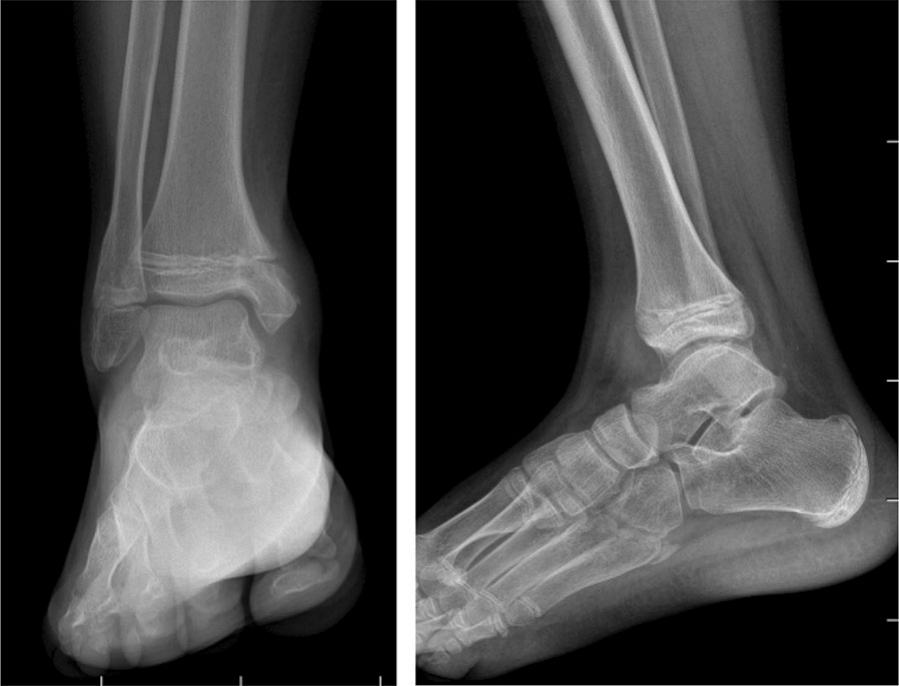
X-rays after implant removal 6 months after operation

**Fig. 4:**
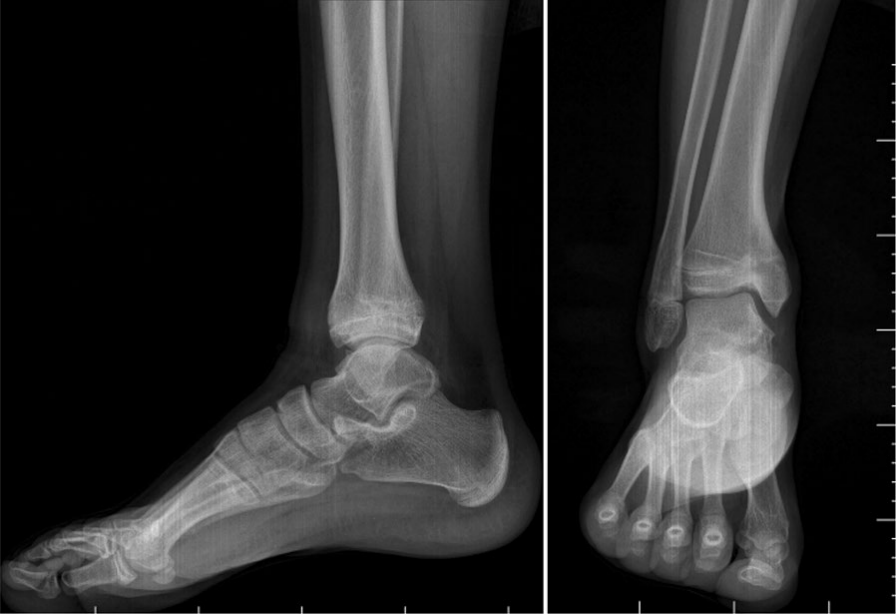
Two-year post-operative X-ray

Patients were grouped according the Salter-Harris classification system (Wuerz and Gurd 2013; Kay and Matthys 2001). Specific injuries, such as tillaux and triplane fractures, were also noted (Wuerz and Gurd 2013). The time until cast removal, time taken to regain full range of motion (ROM), radiological and clinical fracture healing time, and time until full weight bearing were recorded for each patient. The American Orthopaedic Foot and Ankle Society (AOFAS) score was used for clinical assessment. Reduction criteria were determined on the basis of x-ray and CT scan. Implant removal was possible after an average of 6 months after surgery (Figs. 3, 4). Unsatisfactory reduction criteria were determined as the presence of either an intraarticular gap ≥2 mm or a step-off ≥2 mm.

Analysis was performed using SPSS statistical software version 18.0 (SPSS Inc., Chicago, Illinois). Non-parametric tests were used to determine statistical analysis. Mann–Whitney U tests or Kruskal–Wallis tests were performed to test for categorical values, depending on the sample size. p < 0.05 was considered to be significant.

## Results

Seven patients were classified as Salter Harris type 2, 12 as type 3, and 5 as type 4. Additionally, 2 of the type 3 patients were classified as having a tillaux fracture, and 2 patients of type 4 were classified as having a triplane fracture. Cannulated screws were selected as the surgical fixation method for 13 patients, and headless cannulated compressive screws were used for the remaining 11 patients. Seven patients required distal fibular fracture stabilization using K-wire. All fractures were minimally displaced.

The mean follow-up time was 13 months (range 6–38). The mean time until cast removal was 3.5 weeks (range 2–5). A full ROM was achieved on average 5.7 weeks postoperatively (range 4–8). The radiologic healing time was 6.1 weeks (range 4–7). The mean clinical healing time was 6.8 weeks (range 5–8). All patients were allowed full weight-bearing at the end of this period.

The difference between the radiologic healing times for headless compressive screws and cannulated screws used for fixation was not statistically significant (p = 0.487). Likewise, the differences in clinical healing times between fixation methods were not statically significant (p = 0.192). The complication rate among the two groups was statistically similar (p = 0.519) (Table 1).

**Table 1:** Comparison of two screw types

	Cannulated screw	Headless compressive screw	p
Number	13	11	
Age	12,923	11,545	
Radiologic healing time	6.142	6	0.487
Clinical healing time	7.142	6	0.192
AOFAS score	95,384	97,636	0.467
Number of complications	3	2	0.519

The mean AOFAS score was 96 points (range 83–100). The differences in AOFAS scores between the two groups were not statically significant (p = 0.467) (Table 1). In total, 19 patients were totally free of pain, and only 5 had minimal pain during weight-bearing.

Several complications were encountered in 5 patients. Soft tissue infection was detected in only one patient and was treated with oral antibiotics. One patient with a type 2 open fracture required skin grafting after bone fixation. Premature physeal arrest was detected in 3 patients. One case of physeal arrest was noticed in each of the 5th, 6th and 9th months. There were no clinical sign of physeal arrest. Diagnosis was made on the basis of radiographical examination.

## Discussion

Physeal ankle fractures in children must be evaluated by considering the size of the displacement, the growth plate injury, management, and physeal growth remaining (Wuerz and Gurd 2013; Caterini et al. 1991; Barmada et al. 2003; Bible and Smith 2009). Management of these fractures is crucial to preventing early and late complications. Non-displaced physeal fractures can be successfully treated with conservative management (Kay and Matthys 2001). However, displaced or minimally displaced fractures can cause physeal growth, arrest, and deformity of the extremities (Wuerz and Gurd 2013; Kling et al. 1984; Barmada et al. 2003). Surgical treatment should be used for fractures that are displaced by greater than 2 mm and those that have greater than a 1 mm translation of fractures, such as triplane (Feldman et al. 1995) and tillaux fractures (Wuerz and Gurd 2013; Podeszwa et al. 2008; Kling et al. 1984; Caterini et al. 1991; Barmada et al. 2003; Bible and Smith 2009; Jones et al. 2003; Castellani et al. 2009). Salter Harris type 2, 3, and 4 fractures were evaluated in this study. These fracture types have an unstable displaced fragment and require screw fixation after reduction. Surgical intervention of unreduced Salter-Harris type 2, 3, and 4 fractures has been found to decrease growth arrest rate and deformity (Wuerz and Gurd 2013; Lintecum and Blasier 1996; Bible and Smith 2009). Fixation of these fractures also allows early cast removal and rehabilitation.

Meticulous examination is essential to achieve the correct diagnosis of physeal fractures around the ankle. We obtained plain radiographs for all the patients with traumatic ankle injuries by using the Ottawa ankle rules (Stiell et al. 1995). Bisset has revealed that diagnostic errors in pediatric fractures in the form of misses or overcalls occur in 2.7% of the radiographs (Bisset and Crowe 2014). Misses and overcalls are most common in the ankle (Bisset and Crowe 2014). We used computed tomography of the ankle if there was any suspicion of fracture. We also used CT to decide upon and plan operative treatment. Liporace has reported that the use of CT does not significantly change the impression of the amount of displacement per case and further results in patients’ reassignment from non-operative management to operative treatment (Liporace et al. 2012). We recommend taking CT scans of suspicious physeal fractures and displaced fractures before operative planning.

Ensuring the anatomic reduction of epiphyseal fractures is a very demanding procedure. Lintecum and Blaiser (Lintecum and Blasier 1996) have described a method of focusing on direct visualization for open reduction and internal fixation (ORIF). They have reported good results with the anterior surgical approach and percutaneous screw fixation method (Lintecum and Blasier 1996). Castellini has reported a method using Kirschner wires as joysticks to manipulate fractures that are difficult to reduce (Castellani et al. 2009). Here, we used closed reduction and Kirschner wires to assist in manipulation. We achieved a satisfying reduction with the percutaneous Kirschner wire-assisted method, as determined via fluoroscopy. The percutaneous reduction and fixation method resulted in less scar formation than did the open method.

Different types of implants are used for the fixation of epiphyseal fractures. Kirschner wires, smooth pins, tension band fixation, metallic screws, and bioabsorbable screws have all been used for fixation in previous studies (Wuerz and Gurd 2013; Podeszwa et al. 2008; Castellani et al. 2009; Sankar et al. 2013). Kirschner wires and smooth pins cannot be used for compression, but all the others are useful for compression. A recent biomechanical study has demonstrated that metallic screw fixation in the distal tibia significantly alters the articular pressure in the ankle joint (Charlton et al. 2005). A comparison of bioabsorbable screws and metallic screws for the distal tibial physeal fracture has demonstrated similar results for each screw type (Podeszwa et al. 2008). Here, we compared cannulated screws with headless cannulated screws in distal tibial physeal fractures. Screws were fixed parallel to the physis and articular surface while remaining within the epiphysis (Wuerz and Gurd 2013; Castellani et al. 2009). We inserted the cannulated screws percutaneously, fixing them parallel to the physis. None of the screws penetrated the physeal plate in our method, and we were able to achieve intraarticular fixation safely.

Percutaneous screws have been used for the fixation of epiphyseal ankle fractures in many studies in the literature (Podeszwa et al. 2008; Castellani et al. 2009; Sankar et al. 2013; Charlton et al. 2005; Podeszwa and Mubarak 2012). Lintecum and Blaiser have reported good clinical results after using percutaneous cannulated screws (Lintecum and Blasier 1996). Crawford has reported success by fixing percutaneous cannulated screws in tillaux and triplane fractures (Crawford 2012). Podezswa has demonstrated that bioabsorbable screws lead to similar outcomes (Podeszwa et al. 2008). In this technique, screw removal is not required. In this study, we used 3-mm headless compression cannulated screws and 3.5-mm cannulated screws. Although radiologic healing time and clinical healing time were better in the headless compressive screw group, the difference was not statistically significant. There were no significant differences between headless compression screws or standard cannulated screws in radiologic fracture healing time (p = 0.487) or clinical healing time (p = 0.192), even after accounting for age and fracture type, the type of implant used.

The AOFAS scoring scale is commonly used for ankle fractures. Although the AOFAS score has subjective components, it is still commonly used for orthopedic assessment of the ankle and the foot. The subjective components of this rating scale provide quality of life information that conveys acceptable validity regarding conditions affecting the foot and ankle (Ibrahim et al. 2007). A recent study has compared the psychometric properties of AOFAS and SEFAS (self-reported foot and ankle score) and has found similar results between the two scales (Cöster et al. 2014). We found a mean AOFAS score of 96 in our patients, thus indicating a good clinical result. The single patient who did have a low AOFAS score (83) had had a motor vehicle accident and presented with an open injury. High-energy trauma is a risk factor for poor clinical outcomes (Leary et al. 2009).

Epiphyseal ankle fractures can result in several complications. Early and late-term complications were encountered in 5 (20.8%) patients. Two patients had open fractures at the time of arrival at the emergency room. One patient needed skin grafting. We observed 3 (12.5%) premature physeal arrests, 2 of which were Salter Harris type 4 and 1 was type 3. The incidence of premature physeal closure varies by fracture type, with closure in 2–40% of Salter Harris type 1 and 2 fractures and in 8–50% of type 3 and 4 fractures. Premature physeal closure causes growth disturbance (Barmada et al. 2003). Leary has demonstrated that high-energy trauma is more likely to cause growth arrest than low-energy trauma or sports-related injuries (Leary et al. 2009). In our study, 2 patients with premature physeal closure were in motor vehicle accidents, and 1 suffered a sports-related trauma. The complications therefore do not reflect negatively on the clinical results of this study.

The limitations of this retrospective study were the small number of patients and the short follow-up time. The differences between the performance of the cannulated screw and the headless compressive screw could be better investigated in detail with a larger number of patients and a longer-term follow-up.

## Conclusions

The treatment of physeal ankle fractures is of special concern. Insufficiently treated or untreated physeal fractures have high complication rates in growing children. We demonstrated good clinical results with closed reduction assisted by Kirschner wires and with the percutaneous screw fixation method. Both cannulated and headless compressive screws can be used safely as treatment methods in physeal ankle fractures, and both have satisfactory clinical outcomes.

## References

[CR1] Barmada A, Gaynor T, Mubarak SJ (2003). Premature physeal closure following distal tibia physeal fractures: a new radiographic predictor. J Pediatr Orthop.

[CR2] Bible JE, Smith BG (2009). Ankle fractures in children and adolescents. Tech Orthop.

[CR3] Bisset GS, Crowe J (2014). Diagnostic errors in interpretation of pediatric musculoskeletal radiographs at common injury sites. Pediatr Radiol.

[CR4] Castellani C, Riedl G, Eberl R, Grechenig S, Weinberg AM (2009). Transitional fractures of the distal tibia: a minimal access approach for osteosynthesis. J Trauma.

[CR5] Caterini R, Farsetti P, Ippolito E (1991). Longterm followup of physeal injury to the ankle. Foot Ankle.

[CR6] Charlton M, Costello R, Mooney JF, Podeszwa DA (2005). Ankle joint biomechanics following transepiphyseal screw fixation of the distal tibia. J Pediatr Orthop.

[CR7] Cöster MC, Rosengren BE, Bremander A, Brudin L, Karlsson MK (2014). Comparison of the Self-reported Foot and Ankle Score (SEFAS) and the American Orthopedic Foot and Ankle Society Score (AOFAS). Foot Ankle Int.

[CR8] Crawford AH (2012). Triplane and Tillaux fractures: is a 2 mm residual gap acceptable?. J Pediatr Orthop.

[CR9] Dias LS, Giegerich CR (1983). Fractures of the distal tibial epiphysis in adolescence. J Bone Joint Surg [Am].

[CR10] Feldman DS, Otsuka NY, Hedden DM (1995). Extra-articular triplane fracture of the distal tibial epiphysis. J Pediatr Orthop.

[CR11] Gönç U, Kayaalp A (2004). Ankle fractures in children and adolescents. Acta Orthop Traumatol Turc.

[CR12] Ibrahim T, Beiri A, Azzabi M, Best AJ, Taylor GJ, Menon DK (2007). Reliability and validity of the subjective component of the American Orthopaedic Foot and Ankle Society clinical rating scales. J Foot Ankle Surg.

[CR13] Jones S, Phillips N, Ali F, Fernandes JA, Flowers MJ, Smith TW (2003). Triplane fractures of the distal tibia requiring open reduction and internal fixation: pre-operative planning using computed tomography. Injury.

[CR14] Kay RM, Matthys GA (2001). Pediatric ankle fractures:evaluation and treatment. J Am Acad Orthop Surg.

[CR15] Kling TF, Bright RW, Hensinger RN (1984). Distal tibial physeal fractures in children that may require open reduction. J Bone Joint Surg Am.

[CR16] Leary JT, Handling M, Talerico M, Yong L, Bowe JA (2009). Physeal fractures of the distal tibia: predictive factors of premature physeal closure and growth arrest. J Pediatr Orthop.

[CR17] Lintecum N, Blasier RD (1996). Direct reduction with indirect fixation of distal tibial physeal fractures: a report of a technique. J Pediatr Orthop.

[CR18] Liporace FA, Yoon RS, Kubiak EN, Parisi DM, Koval KJ, Feldman DS, Egol KA (2012). Does adding computed tomography change the diagnosis and treatment of Tillaux and triplane pediatric ankle fractures?. Orthopedics..

[CR19] Mizuta T, Benson WM, Foster BK, Paterson DC, Morris LL (1987). Statistical analysis of the incidence of physeal injuries. J Pediatr Orthop.

[CR20] Peterson CA, Peterson HA (1972). Analysis of the incidence of injuries to the epiphyseal growth plate. J Trauma.

[CR21] Podeszwa DA, Mubarak SJ (2012). Physeal fractures of the distal tibia and fibula (Salter-Harris type I, II, III, and IV fractures). J Pediatr Orthop.

[CR22] Podeszwa DA, Wilson PL, Holland AR, Coplay LA (2008). Comparison of bioabsorbable versus metallic implant fixation of physeal and epiphyseal fractures of the distal tibia. J Pediatr Orthop.

[CR23] Sankar B, Lee NY, Henman PD (2013). Periosteal tension band fixation of a pronation external rotation type fracture of the ankle in a child. Orthopedics..

[CR24] Stiell I, Wells G, Laupacis A, Brison R, Verbeek R, Vandemheen K, Naylor CD (1995). Multicentre trial to introduce the Ottowa ankle rules for use of radiography in acute ankle injuries. Multicentre ankle study group. BMJ..

[CR25] Worlock P, Stower M (1986). Fracture patterns in Nottingham children. J Pediatr Orthop.

[CR26] Wuerz TH, Gurd DP (2013). Pediatric physeal ankle fracture. J Am Acad Orthop Surg.

